# Role of heparin in pulmonary cell populations in an in-vitro model of acute lung injury

**DOI:** 10.1186/s12931-017-0572-3

**Published:** 2017-05-10

**Authors:** Marta Camprubí–Rimblas, Raquel Guillamat-Prats, Thomas Lebouvier, Josep Bringué, Laura Chimenti, Manuela Iglesias, Carme Obiols, Jessica Tijero, Lluís Blanch, Antonio Artigas

**Affiliations:** 1 Institut d’ Investigació i Innovació Parc Taulí (I3PT), Sabadell, Spain; 2 Universitat Autonoma de Barcelona, Bellaterra, Catalunya Spain; 3 CIBER de Enfermedades Respiratorias (CIBERES), Sabadell, Spain; 4 Intensive Care Unit, Ponchaillou University Hospital, Rennes, France; 5 U991 INSERM Unit, Rennes, France; 6 Department of Thoracic Surgery, Hospital Universitari Mutua Terrassa, University of Barcelona, Barcelona, Spain; 7 Critical Care Center, Corporació Sanitària i Universitària Parc Taulí, Sabadell, Spain; 8 Fundació Parc Taulí, C/Parc Taulí 1, 08208 Sabadell, Spain

**Keywords:** Acute Respiratory Distress Syndrome (ARDS), Alveolar macrophages, Alveolar cells, Fibroblasts, Anticoagulants, Inflammation

## Abstract

**Background:**

In the early stages of acute respiratory distress syndrome (ARDS), pro-inflammatory mediators inhibit natural anticoagulant factors and initiate an increase in procoagulant activity. Previous studies proved the beneficial effects of heparin in pulmonary coagulopathy, which derive from its anticoagulant and anti-inflammatory activities, although it is uncertain whether heparin works. Understanding the specific effect of unfractioned heparin on cell lung populations would be of interest to increase our knowledge about heparin pathways and to treat ARDS.

**Methods:**

In the current study, the effect of heparin was assessed in primary human alveolar macrophages (hAM), alveolar type II cells (hATII), and fibroblasts (hF) that had been injured with LPS.

**Results:**

Heparin did not produce any changes in the Smad/TGFß pathway, in any of the cell types evaluated. Heparin reduced the expression of pro-inflammatory markers (TNF-α and IL-6) in hAM and deactivated the NF-kß pathway in hATII, diminishing the expression of IRAK1 and MyD88 and their effectors, IL-6, MCP-1 and IL-8.

**Conclusions:**

The current study demonstrated that heparin significantly ameliorated the cells lung injury induced by LPS through the inhibition of pro-inflammatory cytokine expression in macrophages and the NF-kß pathway in alveolar cells. Our results suggested that a local pulmonary administration of heparin through nebulization may be able to reduce inflammation in the lung; however, further studies are needed to confirm this hypothesis.

## Background

Acute respiratory distress syndrome (ARDS) is a common and devastating illness characterized by lung inflammation, endothelial and epithelial injury, increased vascular permeability and oedema; all of these factors lead to organ dysfunction. ARDS results from a direct injury such as a pneumonia or aspiration of gastric contents or from a systemic injury such as sepsis or severe trauma [1–4].

Current therapeutic strategy to decrease ARDS-associated mortality is to utilize protective mechanical ventilation in combination with low tidal volume. However, morbidity and mortality remain high, both exceeding 40% [5–7]. The need for new specific pharmacological therapies has carried to examine the role of altered coagulation and fibrinolysis in the pathogenesis of ARDS.

Pulmonary coagulopathy is intrinsic to ARDS and directly dependent on the severity of acute lung injury (ALI) and linked to the outcome of ARDS. This disease is characterized by the coagulation cascade activation and reduced fibrinolysis that leads to fibrin deposition in the airspaces and triggers inflammation. All of this occurs because of the alveolar type II cell damage and also the pro-inflammatory activation of macrophages.

In recent years, several preclinical studies have supported the use of nebulized or systemic anticoagulants to prevent and treat ALI in diverse animal models [8, 9]. Moreover, numerous clinical trials were performed with patients with ARDS or sepsis requiring unfractionated heparin (UFH), although results have been conflicting. High dose of nebulized heparin decreased pulmonary coagulation in the lung of patients with ARDS without causing systemic bleedings [10]. Furthermore, patients treated with nebulized heparin were ventilated with lower tidal volumes compared to control patients during the first 7 days of ventilation; determining an improvement in the lung function that was associated with the dosage and the days that the nebulized heparin was administered. However the administration of heparin was ineffective in the improvement of patient’s outcomes such as the survival at day 28 [11–15]. Moreover, nebulized heparin combined with N-acetylcysteine for burn inhalation injury decreased lung injury scores and the duration of mechanical ventilation. Besides, heparin was tested as a treatment in other lung diseases such as cystic fibrosis with favourable results [16].

Cellular and molecular mechanisms that control intra-alveolar fibrin deposition are not completely understood. The modulation of fibrin deposition, coagulation and fibrinolysis could be important targets in ARDS treatment. The use of anticoagulants or/and antithrombotic agents such as heparin could ameliorate lung pathology. Inhaled heparin was administered in healthy patients with no side effects [17]. Elucidate the pathways involved in the injury improvement will allow us to look for new therapeutically strategies. As well, cell-cell interaction during ARDS worsens the damage and to design new target treatments for each specific cell are need.

It is known that heparin has anti-inflammatory as well as anticoagulant effects, though the mechanism is unclear. Determine the cross-talk between coagulation and inflammation pathways that are modified by heparin would be useful for ARDS treatment.

The objective of this study was to test the effect of UFH, in inflammation–proliferation–permeabilisation in various primary lung cells such as human alveolar macrophages (hAM), human alveolar type II cells (hATII), and human fibroblasts (hF) in vitro after lipopolysaccharide (LPS)-induced acute injury. Effects on the inflammatory response and potential mechanisms such as the NF-kß and Smad/TGFß pathways were investigated.

## Methods

### Ethics statement

Fourteen biopsies from human lungs for isolation of primary human alveolar macrophages (hAM), fibroblasts (hF) and alveolar type II cells (hATII) were obtained from patients that were submitted to a lobectomy and had previously provided informed consent. The biopsies were obtained from distal areas of the tumour and were histologically normal. Patients did not have interstitial diseases, they were not smokers in the last 2 years and they were between 55 and 75 years old. This study was reviewed and approved by the respective local Ethics Committee.

### Isolation of alveolar macrophages

hAM were obtained by broncho-alveolar lavage (BAL), which was performed with 50 ml of sterile saline (0.15 M; 0.9% NaCl). BAL cells were centrifuged (800 x g, 10 min) and the cell pellet with hAM was resuspended in RPMI 1640 medium (Gibco, USA) supplemented with 10% inactivated foetal bovine serum (FBS) (Gibco, USA), 1 mM L-glutamine, penicillin-streptomycin (50 U/ml, 0.05 mg/ml, respectively), 0.025 mg/ml Vancomycin (Pfizer, Spain) and 0.1 mg/ml cefotaxime (Normon, Spain) and 1 mM HEPES. hAM were plated in 24-well plastic dishes (2x10^5^ cells/well) with 1 ml of supplemented medium and precultured for 24 h at 37 °C and 5% CO2.

**Fig. 1:**
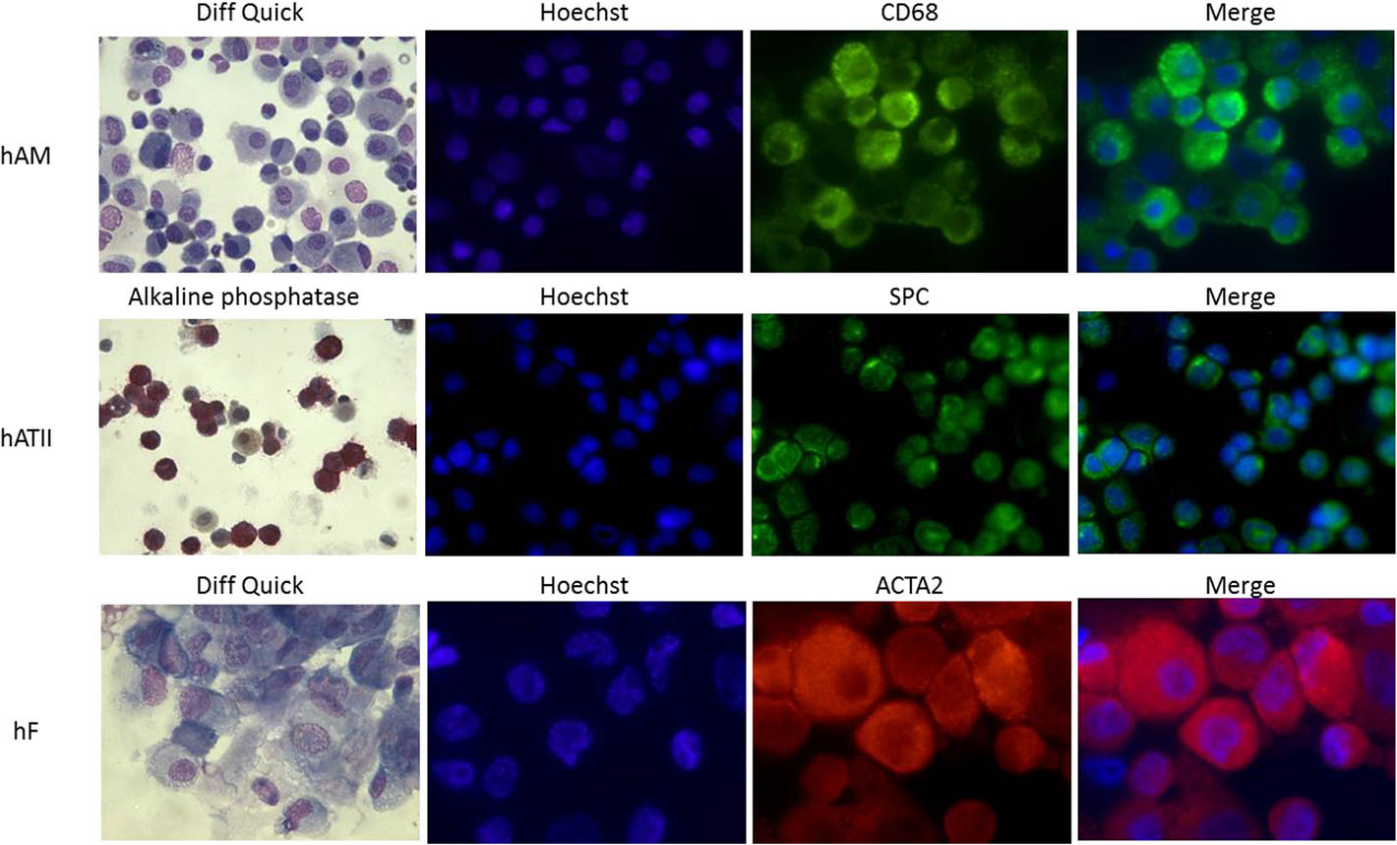
Purity of isolated cells. hAM were stained with Diff quick to differentiate macrophages, neutrophils and lymphocytes and also CD68 immunofluorescence. hATII were stained with alkaline phosphatase and also with Surfactant C protein immunofluorescence. hF were stained with Diff Quick and ACTA2 immunofluorescence. Diff quick and alkaline phosphatase images had x400 magnification. Images of Hoechst, CD68, SPC and ACTA2 immunofluorescences had x600 magnification (hAM: human alveolar macrophages; hATII: human alveolar type II cells; hF: human fibroblasts, SPC: surfactant C protein and ACTA2: alpha smooth actin)

To estimate the purity of isolated hAM, cytospin preparations were stained using the Diff-Quick kit (Diagnostics Grifols, Spain), according to the manufacturer’s protocol. To reinforce the purity of hAM, preparations were fixed in 4% paraformaldehyde and blocked with a solution of PBS, 3% FBS and 1% BSA for 2 h at room temperature. hAM were incubated overnight with mouse anti-rat CD68 (1:100) (Acris Antibodies, USA), washed with PBS and incubated for 1 h at 37 °C with goat anti-mouse IgG-FITC (1:500) (Santa Cruz Biotechnology, USA). After a PBS 1X lavage, cells were incubated 5 min with HOECHST (1:1000) (Invitrogen, USA) (Fig. 1).

### Isolation of human alveolar epithelial type II cells

hATII cells were isolated from lung biopsies after digestion using 0.25% of trypsin type I (Sigma, Germany) in Hanks Balanced Salt solution (HBSS) (Gibco, USA) at 37 °C for 20 min. Once digested, the tissue was chopped in the presence of albumin (Grifols, Spain) and DNase (250 μg/ml) (Roche, Germany). The cell suspension was filtered through 100- and 40-μm nylon meshes, then centrifuged with a density gradient of 1.077 g/ml of Lypmphoprep solution (Sigma, Spain), at 600 x g for 25 min. The interface containing ATII cells and interstitial macrophages was collected, mixed with DNAse (100 μg/ml) and centrifuged at 600 x g for 15 min. The pellet was then resuspended in DNAse (100 μg/ml) and incubated on Petri dishes for 1 h at 37 °C to remove the remaining interstitial macrophages. Unattached cells were hATII cells, which were collected and seeded at a density of 2 × 10^5^ on 24-well plastic dishes, in 1 ml of supplemented DCCM1 (10% FBS (Gibco, USA), 1 mM L-glutamine, penicillin-streptomycin (50 U/ml, 0.05 mg/ml, respectively), μg/ml Vancomycin and cefotaxime and 1 mM HEPES). The cells were precultured for 48 h at 37 °C and 5% CO2.

The purity of isolated hATII was measured by the presence of intracellular alkaline phosphatase (Sigma,. Spain). Further, hATII cells were fixed in paraformaldehyde, and the immunofluorescence protocol described for hAM was performed. Antibodies used were mouse anti-rat SPC (1:100) (Santa Cruz Biotechnology, USA) and goat anti-mouse IgG-FITC (1:500) (Santa Cruz Biotechnology, USA) (Fig. 1).

### Isolation of fibroblasts

hF were obtained from lung explants. Lung slices (1 mm) were cut and placed in Petri dishes of 90 mm^2^. The small pieces were separated by 2–3 cm and incubated with RPMI 1640 medium (Gibco, USA) supplemented with 10% inactivated foetal bovine serum (FBS) (Gibco, USA), 1 mM L-glutamine, penicillin-streptomycin (50 U/ml, 0.05 mg/ml, respectively), 0.025 mg/ml Vancomycin (Pfizer, Spain) and 0.1 mg/ml cefotaxime (Normon, Spain) and 1 mM HEPES. The explants were removed 21 days later, and fibroblasts grew until confluence. Fibroblasts were used between passages 2 and 7. The cells at 85% confluence were trypsinised (Sigma, Spain) and seeded at a density of 5 × 10^4^ on 24-well plastic dishes.

HF cytospin preparations were stained with Diff-Quick kit following manufacturer’s protocol. Moreover, hF cells were fixed in paraformaldehyde and the immunofluorescence protocol described above was performed. Antibodies used were mouse anti-rat ACTA2 (1:100) (Proteintech, USA) and goat anti-rabbit IgG-TR (1:500) (Santa Cruz Biotechnology, USA) (Fig. 1).

### Injury induced by LPS and treatment with heparin

All cell types were exposed to Lipopolysaccharide from *Escherichia coli* 055:B5 (LPS) alone (hAM: 50 ng/ml, hATII: 50 μg/ml and hF: 50 μg/ml) (Sigma, Spain) in serum-free media or in combination with unfractionated sodium heparin (0.1 IU/ml) (Hospira Products Farmac, Spain), which was added 2 h after the LPS exposure. A control of untreated cells and a control with heparin alone were established. hAM were collected 7 h after heparin addition with 500 μl of TRIzol reagent (Ambion, USA) and frozen at −80 °C. hATII cells and hF were collected 24 h after heparin administration. Heparin optimal dose was established from literature [18] and previous studies performed in our laboratory. A time course study determined the maximal efficacy point, which was used to perform our analysis.

### RNA isolation and real-time PCR analysis

Total RNA was extracted from isolated cells using chloroform, isopropanol and ethanol. The optical density at 260 nm and the ratio 260 nm/280 nm were measured with spectrophotometerND-1000 (Nanodrop, USA) to determine the RNA concentrations. Total RNA was reverse-transcribed into cDNA according to the Reverse Transcriptase Core kit (Eurogentec, Belgium), using Alpho-SC (Analytikjena, Germany) thermocycler. PCR amplification was performed in 7500 RealTime PCR System (Applied Biosystems, USA) using SYBR green (Kapa Biosystems, Germany) and the corresponding human primers (Table 1). The PCR started at 95 °C for 10 min, followed by 40-cycle amplification (15 s at 95 °C, 60 s at 60 °C and 2 min at 72 °C). Data are shown as target gene expression relative to GAPDH and fold over Control group; ΔΔCt method was used to correct all the PCR data.

**Table 1:** List of primers and their corresponding sequences used for PCR analysis

Gene	Forward primer	Reverse primer
GAPDH	5′ GAT CAT GAG CAA TGC CTC CT 3′	5-TGT GGT CAT GAG TCG TTC CA-3
TNF-α	5′TCC TTC AGA CAC CCT CAA CC 3′	5-AGG CCC CAG TTT GAA TTC TT-3
IL8	5′ ATTTCTGCAGCTCTGTGTGAAGGTGC 3′	5 -TTGTGGATCCTGGCTAGCAGA C-3
MCP1	5′ CAAACTGAAGCTCGCACTCTCGCC 3,	5 -ATTCTTGGGTTGTGGAGTGAGTGTTCA-3
IRAK1	5′ CCAGCCCCTTCTTCTACCAA 3′	5′ AGCATACACCGTGTTCCTCA 3′
TGF-β	5′ CGGATCAGCGTTTATCAGGT 3′	5′ CAACTTGGGGTTGATGCTCT3′
Myd88	5′ TCACCACACTTGATGACCCC 3′	5′ CGGCACCTCTTTTCGATGAG 3′
SMAD2	5′ TAAAGTGCCTGGGATTGAGG 3′	5′ GTGTGCCTGGGACTTGTTTT 3′
SMAD3	5′ ATAGGTGCTTTGGGCGTATG 3′	5′ CTGCTATCCAGTCACCAGCA 3′
IL6	5′ TACCCCCAGGAGAAGATTCC 3′	5′ TTTTCTGCCAGTGCCTCTTT 3′

#### Multiplex analysis

Media of all the cells in all treated conditions was collected after the treatment, 7 h in the case of hAM and 24 h in the case of hF and hATII. A 4-plex for TNF-α, IL-6, IL-8 and MCP-1 and a single multiplex for TGF-β was performed with the samples of 4 biopsies. The multiplex were performed following the manufacturer’s protocol (eBioscience, Germany). The results are expressed in pg/ml. The sensivity for TNF-α and IL-6 was 0,4 pg/ml, 1,2 pg/ml per IL-8, 0,6 pg/ml per MCP-1 and 0,96 pg/ml per TGF-β.

#### Immunofluorescence

Cells were seeded in cell chambers (Merck Millipore, Germany) and treated as explained before. After 7 h for hAM and 24 h for hATII and hF, cells were fixed with formalin (4%) during 5 min and permeabilized with PBS + 0,2% Triton during 10 min. After, we incubated cell chambers with blocking solution (PBS with 1% albumin and 3% fetal bovine serum) for 2 h at room temperature. NF-kß (1:200, Ref: CPA9199), IRAK-1 (1:100; Ref: TA305934), MyD88 (1:100; Ref: TA30599) and Smad2/3 (1:200; CPA-1707) antibodies (ACRIS, Spain) were used to determine the protein expression by immunofluorescence in hAM, hATII and hF after all the treatments. The cells were incubated 2 h at room temperature with the primary antibodies. After 3 washes with PBS, slides were incubated with the secondary antibodies for 1 h 30 min at room temperature in the dark. We used anti-mouse-FITC as a secondary per NF-kß, anti-rabbit-FITC as a secondary per IRAK-1 and anti-rabbit-Alexa647 as a secondary per MyD88 and Smad2/3. All the secondary antibodies were used a 1:800 dilution (Santa Cruz, USA). After nucleus were stained with Hoechst (Thermo Fisher, Germany) (1:10000 dillution) for 2 min. Slides were washed again in PBS and mounted with Fluoromount™ Aqueous Mounting Medium (Sigma; USA). Light and fluorescence microscopy were performed using a Nikon Eclipse Ti microscope.

### Flow cytometry

A cell death assay was performed using the Annexin V-FITC and propidium iodide (PI) apoptosis and necrosis detection Kit (Clinisciences, France). All cell types (hAM, hATII and hF) were injured with LPS and treated with heparin, as previously described. Cells were trypsinised (Sigma, Spain), washed with PBS 1X and 2 × 10^7^ cells were resuspended in 1 ml of binding buffer (Clinisciences, France). Ten microliters of Annexin V-FITC solution and 15 μl of PI were added to each treatment condition and incubated for 45 min at 4 °C in the dark. Three hundred microliters of PBS 1X were added, and the stained cells were analysed with flow cytometry (FACSCanto).

### Statistical analysis

Data were expressed as mean ± standard error of the mean (SEM) and the *n* = 8 for all the study groups. All results were analysed by one-way ANOVA with multiple comparisons, and Newman-Keuls post-hoc test was applied. Statistical significance was considered at *p* ≤ 0.05.

## Results

### Purity

Primary cells (hAM, hATII and hF) of human biopsies were obtained with purities of 98, 78 and 100%, respectively (Fig. 1). The main impurities in hATII are caused by macrophages and after the media change at 48 h the major part of the impurities are removed. The hATII purity is similar to the purities obtained in other published papers [19, 20].

### Heparin reduces classical pro-inflammatory cytokines on hAM

**Fig. 2:**
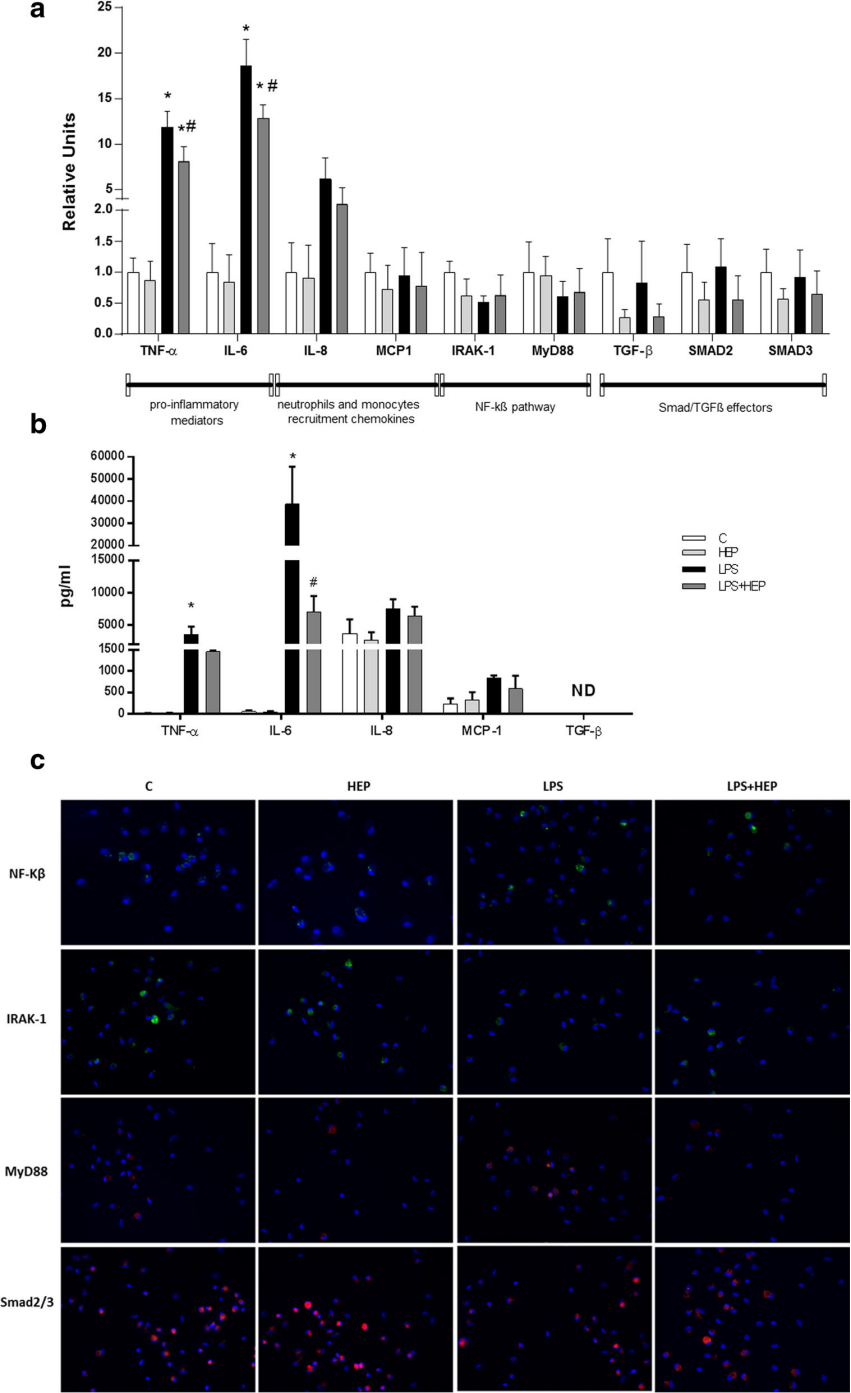
Human alveolar macrophages gene expression. **a** Expression of TNF-α, IL-6, IL-8, MCP-1, IRAK-1, MyD88, TGF-β, Smad2 and Smad3 evaluated by q-PCR at 7 h after LPS treatment. Data are expressed mean ± SEM (ΔCt correction was applied using GAPDH as a housekeeping gene and units are relative to the expression of control group) (*n* = 8 samples per group). **b** Protein expression for TNF-α, IL-6, IL-8, MCP-1 and TGF-β (*n* = 4 samples per group). Data are expressed mean ± SEM. *p ≤ 0.05 vs control groups; #*p* ≤ 0.05 vs LPS group **c** Immunofluorescence for NF-kß, IRAK-1, MyD88 and Smad2/3 and all the treatments are shown. Magnification is 400x. (ND: non-detectable; LPS: Lipopolysaccharide from Escherichia coli 055:B5 and HEP: unfractionated heparin)

The effect of heparin on LPS-injured hAM was assessed after 7 and 24 h by q-PCR. The response of macrophages was observed at 7 h (changes not found at 24 h, data not shown). No changes were observed in the expression of IRAK-1 and MyD88 in different treated hAM. LPS increased significantly the expression of the most common pro-inflammatory mediators of ARDS, IL-6 and TNF-α and heparin reduced them significantly. Moreover, no changes in the expression of monocytes recruitment and neutrophils chemokines were measured. No changes were found in the expression of TGF-β or its effectors (Smad2 and Smad3) in the different hAM groups (Fig. 2a). TNF-α and IL-6 protein expression was also increased by the LPS and after the treatment with heparin was significantly reduced (Fig. 2b). In Fig. 2c we can observe different stainings for NF-kß, IRAK-1, MyD88 and Smad2/3; no differences with the different treatments were perceived.

### Heparin inhibits NF-kß pathway on hATII cells

**Fig. 3:**
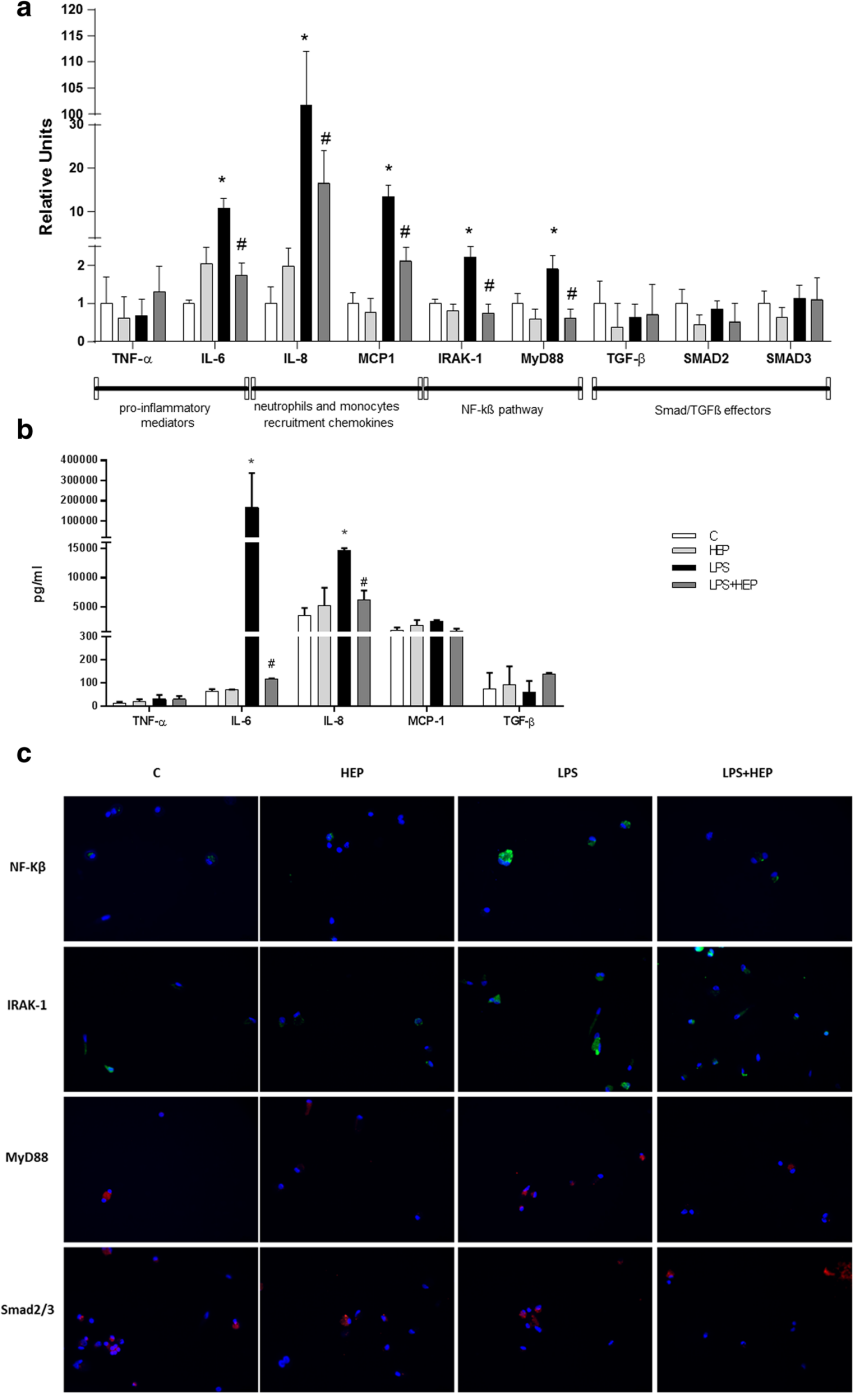
Human alveolar type II cells gene expression. **a** Expression of TNF-α, IL-6, IL-8, MCP-1, IRAK-1, MyD88, TGF-β, Smad2 and Smad3 evaluated by q-PCR at 24 h after LPS treatment. Data are expressed mean ± SEM (ΔCt correction was applied using GAPDH as a housekeeping gene and units are relative to the expression of control group) (*n* = 8 samples per group). **b** Protein expression for TNF-α, IL-6, IL-8, MCP-1 and TGF-β (*n* = 4 samples per group). Data are expressed mean ± SEM. group **c** Immunofluorescence for NF-kß, IRAK-1, MyD88 and Smad2/3 and all the treatments are shown. Magnification is 400x.*p ≤ 0.05 vs control groups; #*p* ≤ 0.05 vs LPS group (LPS: Lipopolysaccharide from Escherichia coli 055:B5 and HEP: unfractionated heparin)

The gene expression produced by hATII cells cultured with LPS and heparin were measured 24 h after heparin administration (no changes were observed at 7 h, results not shown). IRAK-1 and MyD88 levels were increased when cells were injured with LPS, and the addition of heparin diminished their expression significantly. Furthermore, higher levels of IL-6, MCP-1 and IL-8 were observed in the LPS group and heparin reversed those increases significantly. TGF-β, Smad2 and Smad3 did not present any changes when hATII cells were injured by LPS or treated with heparin (Fig. 3a). These mRNA expression results were confirmed by the protein expression. hATII increased their expression of IL-6 and IL-8 after LPS treatment and heparin was able to reduced significantly the levels of this both cytokines (Fig. 3b). However, the effect in MCP-1 observed in mRNA could not be confirmed at protein level. In Fig. 3c, it could be noticed an increase in the labelling for NF-kß, IRAK-1, MyD88 in the LPS treated group compared to the other groups. In the group LPS + HEP we cannot observe any difference compared to control.

### Heparin does not affect NF-kß nor TGF-β pathways on hF

**Fig. 4:**
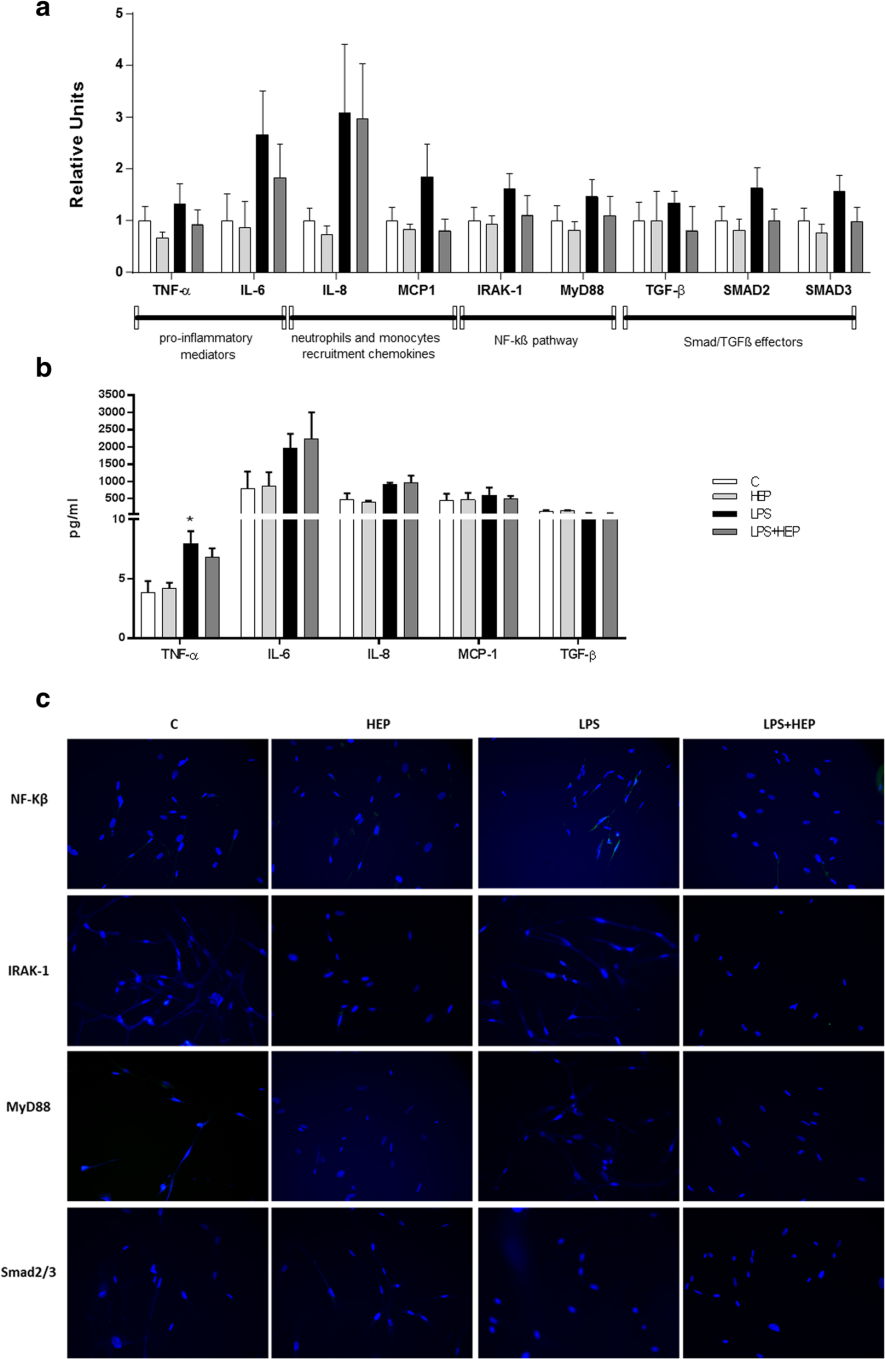
Human fibroblasts gene expression. **a** Expression of TNF-α, IL-6, IL-8, MCP-1, IRAK-1, MyD88, TGF-β, Smad2 and Smad3 evaluated by q-PCR at 24 h after LPS treatment. Data are expressed mean ± SEM (ΔCt correction was applied using GAPDH as a housekeeping gene and units are relative to the expression of control group) (*n* = 8 samples per group). **b** Protein expression for TNF-α, IL-6, IL-8, MCP-1 and TGF-β (*n* = 4 samples per group). Data are expressed mean ± SEM group **c** Immunofluorescence for NF-kß, IRAK-1, MyD88 and Smad2/3 and all the treatments are shown. Magnification is 400x. *p ≤ 0.05 vs control groups; #*p* ≤ 0.05 vs LPS group (LPS: Lipopolysaccharide from Escherichia coli 055:B5 and HEP: unfractionated heparin)

The effect of heparin on LPS-injured hF was examined 24 h after heparin treatment (no changes were observed at 7 h, results not shown). No changes were observed in the expression of IRAK-1 and MyD88 in the different hF groups. An observable increase of IL-6, MCP-1 and IL-8 was observed in the LPS group and reduced by heparin in the case of IL-6 and MCP-1, although the decrease was not significant. No changes were found in the expression of TGF-β or its effectors in the different hF treated groups (Fig. 4a). In protein expression, TNF-α was increased after treatment with LPS, however no other changes were observed in the effectors evaluated (Fig. 4b). In Fig. 4c no labelling for any antibody was observed.

### Apoptosis and Necrosis are not affected by heparin

**Fig. 5:**
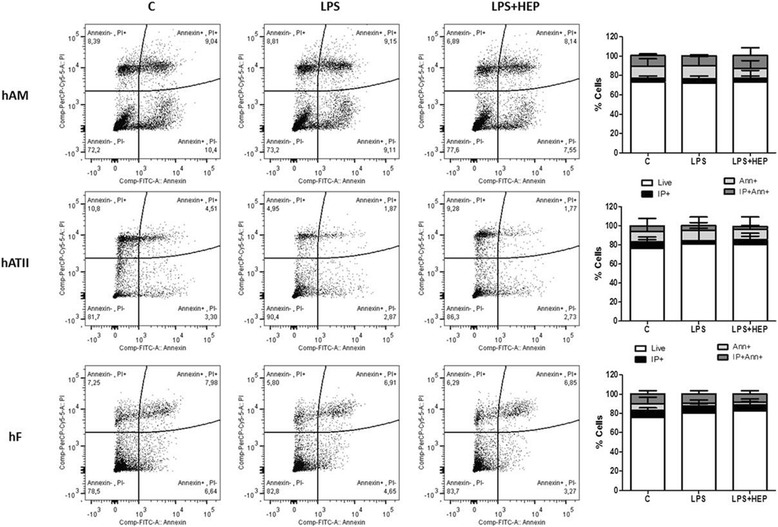
hAM, hATII cells and hF apoptosis/necrosis measured by Annexin V and PI staining with flow cytometry. The proportion of live cells (Annexin V-FITC-/PI-), early apoptotic cells (Annexin V-FITC+/PI-), necrotic cells (Annexin V-FITC-/PI+), late apoptotic/necrotic cells (Annexin V-FITC+/PI+). No changes were observed in LPS treated samples or in LPS + Hep treated ones (hAM:human alveolar macrophages; hATII: human alveolar type II cells; hF: human fibroblasts; FITC: Fluorescein; PI: propidium iodide; LPS: Lipopolysaccharide from Escherichia coli 055:B5 and HEP: unfractionated heparin)

Annexin V/PI assay was performed to detect apoptotic and necrotic cells after LPS injury and heparin treatment. There was no evident increase in apoptosis or necrosis in the LPS-treated group in any of the cell populations studied. Heparin did not produce any change in apoptosis or necrosis (Fig. 5).

## Discussion

In the present study, we demonstrated the specific anti-inflammatory and immunomodulatory effect of heparin in various cell lung populations. We studied primary human macrophages (hAM), alveolar type II cells (hATII) and fibroblasts (hF). Few studies are now focused in the evaluation of infection and inflammation response in specific cell populations. The mechanisms underlying the effects of heparin on the inflammatory response are not fully understood, which is why we decided to study the individual response of human cell lung populations stimulated by LPS after heparin treatment in vitro. In this study we determined that heparin regulates inflammation through the NF-kß pathway. Moreover, all of the previous studies are performed in cell lines and not in primary cells, which is less useful as cell line response is less translational to in vivo results than primary cell response.

Heparin, a natural anticoagulant, is synthesized by various cells, including mast cells [18]. Heparin is broadly applied in the clinics as an anticoagulant drug [21, 22]. Several studies showed controversial results about the anti-inflammatory effects of heparin in in-vivo and in-vitro LPS-induced ALI models [10, 18, 23–26].

When infectious bacteria invade the lung, they activate inflammation that leads to cytokine release and endothelial activation and dysfunction [27]. LPS is a potent inductor of pro-inflammatory factors such as TNF-α and IL-1β as well as IL-8 and CXCL family chemokines that reproduce the effect of an infection [28]. TNF-α is one of the first inflammatory factors released in the inflammatory process, and it plays a key role in the network of inflammatory mediators. There is a significant body of evidence that concentration of TNF-α and IL-6 is increased in BALF of patients with ARDS [29], and the persistent elevation of pro-inflammatory cytokines has been associated with a worse outcome in patients with ARDS or sepsis. However, the inflammatory response is a multi-step process involving different cytokines and chemokines released from numerous and different cells at different time-points.

It is remarkable that macrophages are the first activated cells when an infection happens [30]. Our results showed that heparin effectively inhibits slightly LPS-induced TNF-α, and IL-6 mRNA and also protein expression (Fig. 2) in macrophages suggesting that heparin inhibits the induction of this pro-inflammatory cytokines. These findings suggest that heparin exerts its anti-inflammatory effects by suppressing the production of cytokines and chemokines swiftly in macrophages. The production and release of cytokines occurs more rapidly with macrophages as compared to alveolar cells and fibroblasts, so, small changes in the production of proinflammatory cytokines by these cells could also induce a huge inhibition of inflammation in the lung. So, the quick de-activation of macrophages would be useful to control the classical pro-inflammatory cascade activation.

In case LPS is not rapidly eliminated, other cell types such as alveolar and fibroblasts cells will be activated, which increases inflammation. In addition to classical cytokines, further pathways will be either stimulated, amplifying the inflammatory response. Our findings demonstrate that heparin is able to interrupt the expression of IL-6, MCP-1 and IL-8 by alveolar epithelial cells and also reduce the protein expression in IL-6 and IL-8. MCP-1 is the most important chemokine that regulates migration and infiltration of monocytes and is used for the prediction of prognosis in some diseases as sepsis [31]. IL-8 is a neutrophil chemotactic factor and induces the migration of neutrophils to the site of infection. However the decrease of the expression of MCP-1 protein is not significantly reduced by heparin at this time point. It is known that MCP-1 is regulated and produced after IL-8 secretion, so it could be that at later points a reduction of MCP-1 protein expression might be observed.

It has previously been described that heparin treatment significantly reduces the expression of pro-inflammatory markers, such as TNF-α and IL-1β in lungs of rats with lung injury. This effect was found to be mediated by the inhibition of NF-κB nuclear translocation in the lung. In agreement with these previous observations, we propose that the diminution of the expression of both factors demonstrates an interrupted activation of NF-κB signalling pathway [18]; which is involved directly in the regulation of MCP-1, IL-6 and IL-8. Our results showed correspondingly that the expression of IRAK-1 and MyD88 by the alveolar Type II cells decrease after heparin treatment. MyD88 plays an active role in the phosphorylation and activation of IRAK-1. When IRAK-1’s kinase activity is induced, autophosphorylation occurs and leads to a conformational change that strongly leads to the downstream activation of NF-kß pathway. Here, we show that heparin specifically blocks this pathway in alveolar type II cells, which produce high amounts of MCP-1 and IL-8. Heparin has no effect on IRAK-1 or MyD88 in fibroblasts or macrophages; consequently, NF-kß is not de-activated. The non-regulation of MCP-1 and IL-8 is driven through this pathway.

In our studies, fibroblasts presented higher levels of TNF-α protein at the LPS group, although heparin did not exert any change to this cellular type. Furthermore, any significant change due to the LPS or the heparin treatment was assessed in the other markers evaluated. Lung fibroblasts are usually not taking part in the response to an infection and the secretion of LPS and it seems that the effect of heparin in this cells is not important at this concentrations.

The main limitation of our study is that we checked mRNA levels and the determination of protein expression was difficult due to the lower amounts of cells that we obtained from the human biopsies. It would be interesting the effects on IRAK phosphorylation’s and of some effectors of NF-kß pathway. However, we were not able to perform western blots because the quantity of protein was not enough to perform this technique. So, we quantified some secreted cytokines effectors of NF-kß and Smad/TGFß pathways in media, and the results fitted to the mRNA results. Also, we did several immunostainigs of some NF-kß and Smad/TGFß mediators that help to consider in the mRNa results. Previous studies have also reported that heparin ameliorated the lung injury induced by LPS in rats via inhibition of the TGF-β/Smad pathway [32]. We were unable to verify this claim. The TGF-β/Smad pathway is upregulated in fibroblasts after treatment with LPS and down-regulated after heparin administration, but these trends were not significant. The involvement of other cell types such as endothelial cells or myofibroblasts could explain the changes in TGF-β/Smad pathway in vivo. Further studies should be performed to confirm this.

We also investigated the apoptotic and necrotic processes that might be involved. LPS stimulation at the used concentrations and at 7 h or 24 h did not induce apoptosis in our human primary cells [33]. It is broadly demonstrated that LPS induces apoptosis in in-vivo lung injury models at late phases [33, 34], however we are just working in early stages of the damage and apoptosis or necrosis are not involved at the intervals we studied. Heparin had no effect in apoptosis nor on necrosis.

In recent years, the number of studies evaluating macrophage activation has increased. This phenomenon is often explored in acute lung injury models. We demonstrated that the role of macrophages is certainly important in the first steps of the inflammatory response; however, alveolar type II cells have more relevance in the maintenance of this response because of NF-kß activation. Additionally, hATII cells might be the responsible in the recruitment of new pro-inflammatory cells, so therapies addressed to regulate these processes may target hATII cells. Nevertheless, in-vivo the crosstalk with different cells could modify the effects found in vitro. New studies with human primary cells and co-cultures evaluating the specific response to infection and inflammation and the crosstalk between cells are necessary to increase the knowledge and improve the targets for new therapies.

## Conclusions

In conclusion, the current study demonstrated that heparin significantly ameliorated the cells lung damage induced by LPS through the inhibition of pro-inflammatory cytokine expression in macrophages and blocking NF-kß pathway in alveolar cells, consequently reducing the production of some of this pathway effectors such as IL-8 and IL-6. Our results suggested that a local pulmonary administration of heparin through nebulization may be able to reduce inflammation in the lung; however, further studies are needed to confirm this hypothesis.

## Abbreviations

ALI: Acute lung injury
ARDS: Acute respiratory distress syndrome
BAL: Broncho-alveolar lavage
FBS: Fetal bovine solution
hAM: Human alveolar macrophages
hATII: Alveolar type II cells
HBSS: Hanks Balanced Salt solution
hF: Fibroblasts
LPS: Lipopolysaccharide from Escherichia coli 055:B5
UFH: Unfractionated heparin
